# Clinical Evolution, Outcomes, and Emerging Preservation Technologies in Pancreas and Islet Transplantation

**DOI:** 10.3390/jcm15176774

**Published:** 2026-08-31

**Authors:** Maria Irene Bellini, Vassilios Papalois

**Affiliations:** 1 Department of Surgery, Sapienza Università di Roma, 00161 Rome, Italy; 2 Department of Surgery and Cancer, Imperial College, London SW7 2AZ, UK

**Keywords:** pancreas transplantation, islet transplantation, hypoimmune cells, machine perfusion

## Abstract

Historically regarded as competitive modalities, pancreas and islet transplantation are increasingly recognized as complementary approaches to beta-cell replacement therapy. While whole-organ pancreas transplantation remains the established gold standard, islet transplantation has transitioned into an effective clinical alternative for selected cohorts of patients suffering from type 1 diabetes. This review provides a comprehensive evaluation of the clinical evolution characterizing these therapeutic modalities, tracing the shift from static cold storage (SCS) toward innovative dynamic preservation technologies, and exploring potential markers for predicting clinical outcomes. A focus on immunosuppression in islet transplantation is provided, emphasizing precise, tolerance-inducing strategies and investigating costimulation blockade, regulatory T-cell (Treg)-based therapies, and biomaterial-enabled local immunoprotection, with the aim of facilitating long-term graft survival while mitigating the metabolic and renal toxicities secondary to chronic calcineurin inhibitor exposure. Current evidence related to the transition from conventional SCS to advanced perfusion platforms, namely hypothermic machine perfusion (HMP), oxygenated hypothermic perfusion (HOPE), normothermic ex vivo perfusion (NEVP), and normothermic regional perfusion (NRP) is examined: the literature suggests that dynamic pancreas preservation platforms may improve metabolic recovery and some islet isolation outcomes; however, evidence that HOPE specifically reduces ischemia–reperfusion injury and consistently increases islet yield in the pancreas remains limited. Although normothermic perfusion provides significant utility for real-time viability assessment and graft reconditioning, its widespread clinical implementation remains constrained by technical impediments, notably interstitial edema. Ultimately, preservation efficacy is conceptualized not merely as a discrete preservation option, but as a multidimensional construct integrating biological integrity, graft usability, and recipient clinical outcomes.

## 1. Introduction

Historically, pancreas transplantation has been regarded as the substitutive therapy for beta-cell replacement, while islet transplantation was considered still in an experimental phase, largely due to the superior early clinical outcomes associated with whole-organ grafts [1,2]. This hierarchy has been increasingly challenged by the emerging clinical evidence demonstrating the utility of both approaches in a convergent manner. Pancreas transplantation has achieved sustained improvements in long-term graft survival through refinements in surgical techniques, immunosuppression, and preservation times [3]. Concurrently, islet transplantation has evolved significantly since the implementation of the Edmonton protocol; in selected non-uremic patients with type 1 diabetes, it now achieves clinical outcomes comparable to whole-organ transplantation, including glycaemic control, prevention of severe hypoglycaemia, and medium-term insulin independence [4,5]. Consequently, a growing body of literature recognizes pancreas and islet transplantation not as mutually exclusive alternatives, but as complementary strategies tailored to specific clinical indications, risk profiles, and donor characteristics [4].

A parallel evolution characterizes the research on preservation modalities. While static cold storage, particularly with the University of Wisconsin solution, remains the clinical standard [6,7], evidence questions its adequacy, especially for extended criteria donors (ECDs) or donation after circulatory death (DCD) pancreata intended for islet isolation [8,9]. Furthermore, dynamic preservation technologies now offer mechanisms for oxygen delivery, metabolic support, waste clearance, and real-time viability assessment [10,11]. However, pancreas-specific validation remains heterogeneous: while hypothermic machine perfusion (HMP) is feasible [12], including in a clinical setting [13], it raises concerns regarding edema and uncertain benefit for whole-organ grafts [14]. Notably, recent data from split-pancreas human models showed no definitive superiority of oxygenated HMP over SCS regarding islet functionality or histological integrity, underscoring that technological promise continues to outpace definitive clinical validation [15].

This review aims to delineate the latest evidence on biotech immunosuppression in islet transplantation and on preservation modalities in pancreas donation and identifies which preservation metrics best predict clinically meaningful outcomes. In particular, the current literature interprets preservation success through heterogeneous indices, including graft survival, insulin independence, islet yield, purity, stimulation index, ATP preservation, edema, or the feasibility of utilizing marginal donors [16,17]. Crucially, these endpoints are related but not interchangeable. From a different perspective, preservation efficacy must be considered as a multidimensional framework encompassing biological integrity, transplant usability, and clinically meaningful recipient outcomes, rather than a solitary technical property. 

## 2. The Evolution of Islet Cell Transplantation

The path of islet cell transplantation reflects a shift from experimental procedures to standardized, minimally invasive clinical therapy [18,19]. This maturation has been enabled by three decades of refinement in islet isolation protocols, donor management, and recipient conditioning [20].

In the early 1980s, the primary barriers to success were rudimentary ductal collagenase digestion methods that resulted in variable islet yields and suboptimal engraftment [21,22]. The introduction of the automated method, pioneered by Ricordi et al. (1988) [23], marked a pivotal technological shift, enabling reproducible, high-yield isolation: by the 1990s, the focus expanded toward standardizing processing and optimizing intraportal delivery, with portal vein infusion becoming the dominant clinical route despite recognized limitations for early islet loss and engraftment [24,25].

The real inflection point in the field, however, was the landmark 2000 Edmonton Protocol. By employing a glucocorticoid-free immunosuppressive regimen, comprising low-dose tacrolimus, sirolimus, and daclizumab induction therapy [26] and ensuring an adequate transplantable islet mass (mean islet dose 11,547 IEQ/kg), this protocol achieved sustained insulin independence in approximately 80% of patients at one year [27]. This breakthrough demonstrated that successful beta-cell replacement was clinically attainable, rather than merely theoretical, although durability remains constrained by graft attrition, donor scarcity, and the cumulative toxicity of chronic immunosuppression [28].

Subsequent decades have focused on addressing the limitations observed post-Edmonton. Technical refinements in donor selection, rescue purification, and pre-transplant culture protocols further maximized the utilization of available islet mass. However, longitudinal follow-up revealed that absolute insulin independence is often transient [29]. Consequently, the clinical paradigm has shifted from an exclusive focus on long-term insulin independence to a more nuanced evaluation of ‘clinical success.’ Current benchmarks now prioritize sustained glycaemic control, the prevention of severe hypoglycaemic episodes, and the maintenance of partial graft function.

As we advance into the 2020s, the focus has pivoted toward persistent immunological and engraftment barriers. Contemporary research emphasizes adjunct strategies, including biomaterials for encapsulation [30,31], localized immunomodulation [32,33], and alternative beta-cell sources such as stem cell-derived or hypoimmune engineered islet products [34,35], aiming to improve graft survival while reducing or potentially eliminating the need for chronic systemic immunosuppression [36,37].

This shift has also redirected the field’s immunological focus from short-term insulin independence toward the long-term durability of graft function and glycaemic control. The most used immunosuppression protocols can reduce acute rejection, but long-term graft outcomes remain limited by chronic rejection and therapy-related infectious, renal, and metabolic complications [38,39,40]. Consequently, a hotly debated topic concerns immunosuppression minimization versus true tolerance induction: while the former aims for less toxic maintenance regimens, the latter seeks graft acceptance without lifelong therapy [41].

In islet transplantation, actively studied tolerance strategies include adoptive regulatory T-cell therapy [42], mixed chimerism induced by donor hematopoietic engraftment [43], and biomaterial-based encapsulation/shielding microenvironments [44]; apoptotic donor-cell infusions are also being explored preclinically [45].

Yet, these approaches remain at different stages of maturity: some have shown encouraging preclinical or early-phase clinical signals, but robust, reproducible tolerance in autoimmune human recipients remains unproven, in part because islet grafts must evade both alloimmunity and recurrent beta-cell autoimmunity [46,47].

Table 1 summarizes the biotechnology applications in islet-transplant immunosuppression, aiming to replace broad systemic drugs with targeted biologics, Treg-based therapies, and biomaterial-enabled local delivery or encapsulation that protect grafts while reducing calcineurin inhibitor (CNI) toxicity.

In summary, the biotech direction in islet-transplant immunosuppression is not one replacement drug but a platform shift: from chronic systemic CNI/steroid exposure toward precision biologics, immune tolerance via Tregs, and biomaterial-based local immunoprotection aiming to preserve graft function while reducing toxicity. The strongest consensus is for antibody-based induction and costimulation blockade, while encapsulation and local delivery are the most developed bioengineering routes for eventually reducing or eliminating systemic immunosuppression.

## 3. Technological Advancements in Pancreas and Islet Preservation

The pancreas is highly susceptible to ischemia–reperfusion injury (IRI) occurring in the period between donation and transplantation. The standard preservation method (static cold storage on ice, SCS) only slows tissue deterioration, resulting in discarded donated pancreata rates varying from 25% in the USA [83] up to 50% in Europe and the UK [84]. Furthermore, the acceptance rate is increasingly challenged by the growing utilization of ECD and DCD organs, in parallel with the ageing of the population.

The pancreas is considered especially vulnerable to IRI because even short periods of interrupted blood flow can trigger microcirculatory failure, oxidative stress, inflammation, and acinar cell damage, which then contribute to graft pancreatitis, thrombosis, and early graft loss. There is evidence that the damage occurring is not just a “lack of oxygen”, but rather a cascade involving reperfusion-driven reactive oxygen species (ROS), endothelial activation, and innate immune activation [85].

More specifically, early ischemia reduces oxygen and ATP, disrupting mitochondrial function and cell metabolism [86,87]. Then, reperfusion adds a second hit: reoxygenation generates ROS and amplifies inflammatory signaling [88]. Altogether, the damage in the pancreas is characterized by microcirculatory impairment, endothelial dysfunction, and leukocyte and platelet adhesion, which may worsen vascular obstruction and thrombosis, sterile inflammation [85] driven by damage-associated molecular patterns and cytokines apoptosis and necrosis, with worse tissue injury when preservation is prolonged or suboptimal.

As for the other transplantable organs, cold ischemia time matters; in fact, longer preservation time worsens injury, and reducing cold ischemia substantially limits damage and thrombosis risk. In consideration of the strong sterile inflammatory response during procurement and after reperfusion, potentially also related to early graft pancreatitis and early loss, recent promising protective preservation strategies suggest benefits from pancreas machine perfusion (PMP) [89].

The concept behind perfusion technology relies on circulating preservation fluid through the donor pancreas to deliver oxygen and metabolic support, wash out harmful metabolites, and permit active conditioning of the graft, rather than relying solely on passive cold storage [90]. More broadly, machine perfusion has improved early post-transplant function in other solid organs, particularly the kidneys, where hypothermic machine perfusion reduces delayed graft function compared with static cold storage [91,92].

While SCS still remains the clinical standard, its inability to facilitate active metabolic support or organ resuscitation has necessitated a transition toward dynamic preservation technologies [89]. The evolution timeline of PMP is reported in Table 2. PMP has moved from rudimentary experimental models toward sophisticated platforms, which can be categorized into three primary modalities: hypothermic +/− oxygen delivery machine perfusion (HMP/HOPE), normothermic ex vivo perfusion (NEVP) and normothermic regional perfusion (NRP).

### 3.1. Hypothermic Machine Perfusion (HMP) and Oxygenated HMP (HOPE)

Hypothermic machine perfusion (HMP) remains the most clinically practical approach to pancreas preservation. By keeping the graft at 4–10 °C and supplying continuous or pulsatile flow, HMP reduces the metabolic suppression associated with static cold storage [92,114]. Recent data further suggest that even short durations of HMP can improve graft reperfusion parameters, permitting real-time assessment of vascular resistance and offering a distinct advantage for vulnerable DCD grafts [113].

Additionally, evidence consistently reports benefit from oxygenation during HMP: oxygen carriers such as M101 further reduce ischemia markers and are associated with better metabolic function post-transplant [90].

HOPE increases tissue resynthesis of ATP stores depleted during ischemia, reduces succinate accumulation, and improves reperfusion parameters [115]. Preclinical and human feasibility studies have consistently demonstrated that HOPE suppresses oxidative stress; prevents microvascular degradation, leading to preserved histological integrity; and, crucially for islet transplantation, optimizes subsequent islet yield [106].

There is an ongoing clinical trial denominated “HOPP”, Hypothermic Oxygenated Pancreas Perfusion, currently recruiting in Oxford (UK), with the first patient being already transplanted. In the study, the donor pancreas is perfused for two hours while the recipient undergoes a kidney transplant, followed by the pancreas transplant [13].

Finally, with regard to insulin secretion, a porcine study supports continuous HOPE, as it reports improved biphasic insulin secretion compared with SCS and end-ischemic perfusion [113]; on the other hand, in another previous experimental setting, split-pancreas preservation methods showed similar insulin secretory function across groups, with no discernible functional differences [15].

### 3.2. Normothermic Ex Vivo Perfusion (NEVP)

Normothermic ex vivo perfusion (NEVP) aims to restore the physiological environment for organ preservation, shifting from mere storage to active metabolic support and viability assessment. By maintaining the pancreas at near-normothermic temperatures (34–37 °C), NEVP facilitates ex vivo metabolism and functional activity, effectively serving as a reconditioning platform [108]. This modality provides a critical “window of opportunity” for real-time monitoring of hemodynamic parameters, including flow, pressure, and vascular resistance, alongside metabolic profiling via lactate clearance and glucose-stimulated insulin secretion [110]. Such comprehensive assessment transforms the perfusion circuit into a diagnostic tool, enabling clinicians to objectively evaluate graft viability prior to transplantation.

Despite its significant potential, NEVP remains technically demanding. Early human EVNP studies suggest that the approach can support short-term metabolic assessment of discarded pancreases, including maintained perfusion parameters and detectable insulin secretion, but these reports also emphasize that the method is still primarily an assessment platform rather than an established preservation strategy [98]. Likewise, porcine normothermic perfusion models have shown preserved cellular viability, oxygen consumption, and endocrine/exocrine function under carefully controlled conditions [102,108]. However, the development of significant interstitial edema remains a primary bottleneck, because pancreas perfusion is highly pressure-sensitive and excess tissue water has been linked to poor macroscopic outflow, hemorrhagic change, and concern for downstream islet isolation efficiency or tissue necrosis when protocols are not tightly optimized [99,106]. Current engineering research therefore focuses less on proving metabolic activity per se than on defining perfusion conditions that limit edema and microvascular injury, particularly through low- or pressure-controlled circuits and alternative oxygen carriers or acellular perfusates designed to maintain oxygen delivery while reducing red-cell-associated perfusion stress [102,106].

### 3.3. Normothermic Regional Perfusion (NRP)

In contrast to ex vivo methods, NRP offers an in situ resuscitation strategy for DCDs. By restoring oxygenated blood flow within the donor post-declaration of death but prior to organ retrieval, NRP restores blood flow to the abdominal organs [116]. This approach effectively converts a warm-ischemic DCD pancreas into a near-DBD (donation after brain death) status, significantly improving islet isolation outcomes by minimizing the duration of warm ischemia [117].

While this approach is theoretically advantageous and has shown promise in liver and kidney transplantation, robust, pancreas-specific clinical evidence remains limited. Currently, its application in pancreas transplantation is considered an emerging donor-reconditioning strategy, with outcomes requiring further rigorous evaluation to establish its impact on graft survival and islet isolation efficiency before widespread clinical adoption.

Table 3 provides an overview of the current evidence on pancreas transplant perfusion, including regional perfusion, ex situ machine perfusion (hypothermic and normothermic), oxygenation strategies, preservation solutions, and experimental/clinical outcomes.

The literature also describes the “Two-Layer Method”, i.e., oxygen supplementation for islet isolation and ductal perfusion, a targeted technique that is mainly suitable for islet isolation, not whole-organ grafts [97].

A promising research direction is the shift toward considering perfusion platforms as multifunctional tools rather than mere preservation machines. For pancreas perfusion, the most practical biomarker set is imaging-based flow/perfusion metrics plus oxygenation and injury markers: CT/CEUS/MRI for regional perfusion, Orthogonal Polarization Spectral imaging (OPS) or laser-based methods for microcirculation, and lactate, amylase, lipase, insulin, and C-peptide for functional/injury readout. In pancreas transplantation, these parameters and markers track ischemia, reperfusion injury, and graft quality, although no universally accepted perfusion biomarker has been recognized [119,120,121,122,123,124] (Table 4).

In particular, the literature suggests the following:

- Perfusion CT is used to quantify pancreatic blood flow and related parameters. It has been linked to pancreatitis severity, diabetes-related microcirculatory change and blood-flow measurement accuracy in experimental and clinical settings [119,134].
- Microcirculatory imaging is especially informative in pancreatitis and transplantation: OPS imaging and intraoperative human imaging show reduced capillary perfusion after reperfusion, and those changes correlate with later exocrine/endocrine injury [122,125].
- Tissue oxygenation markers are able to detect early ischemia before systemic markers rise; in a porcine model, pancreatic microdialysis changes in parallel with local tissue oxygen tension, while systemic lactate/amylase/TNF-alpha remains unchanged early on [120].
- Transplant viability assessment currently relies on a mixed panel rather than a single validated biomarker: lactate dehydrogenase, lactate, glucose, amylase, lipase, insulin, and C-peptide are all used or have been proposed, but no universally accepted pancreas-specific standard exists yet [121,123,124,135].
- Inflammation/ischemia markers such as CRP, lactate-to-pyruvate ratio, urinary trypsinogen-2, and serum amylase/lipase have been proposed for postoperative pancreatitis or fistula settings, but uptake into routine practice remains limited [122,126,136].

This conceptual framework reorients the clinical discourse, transitioning from a binary comparison of technologies to a strategic alignment between preservation modality, graft intent, and endpoint selection. Rather than merely evaluating whether a single preservation technique is superior, the purpose is to identify preservation efficacy as a pathway-specific, outcome-linked construct.

## 4. Outcome Prediction

The most reliable perfusion predictors during PMP are flow, vascular resistance, pressure stability, lactate/pH trajectory, edema/weight gain, and injury markers such as amylase; however, as shown above, most evidence is experimental or early feasibility data, not validated clinical cutoffs [135]. Table 5 reports the commonly utilized parameters and biomarkers to predict clinical outcomes.

Evaluating pancreas viability during PMP relies primarily on hemodynamic and metabolic parameters. High blood flow at a stable pressure serves as the clearest indicator of good organ preservation, whereas low tissue flow rates predict reperfusion injury and subsequent impairment of endocrine performance [138]. Stable or decreasing vascular resistance is the other core hemodynamic marker indicating better graft quality [93,135]. During NEVP, overall viability is suggested by positive metabolic readouts, including stable pH, active insulin secretion, and a progressive decline in lactate levels over time [98]. However, significant risk factors continue to threaten graft outcomes, such as excess edema driven by high pressures and weight gain [99,108], as well as prolonged cold ischemia time [98]. Despite the utility of these assessment markers, ex situ pancreas perfusion currently lacks standardized protocols, leaving optimal clinical thresholds for oxygenation, temperature, flows, and perfusate composition yet to be firmly established [139].

## 5. Conclusions and Future Perspectives

The evolution of pancreas and islet transplantation reflects a fundamental paradigm shift: moving away from a binary, alternative framework, where whole-organ transplantation was viewed as the sole definitive standard and islet transplantation as experimental, toward a model of complementary, precision-based beta-cell replacement. This transition is underpinned by synergistic refinements in surgical techniques, tailored immunosuppressive strategies, and the maturation of preservation technologies.

Central to this advancement is the acknowledgment of the preservation efficacy as a multidimensional construct; preservation is no longer a static, time-dependent variable but a dynamic, outcome-linked platform. Technologies such as HMP, HOPE and emerging normothermic modalities (NEVP/NRP) have transformed the perfusion circuit from a simple storage pump into an active diagnostic and reconditioning tool. Simultaneously, the immunological landscape has pivoted from broad, systemic CNI-based maintenance to targeted, tolerance-inducing strategies, including costimulation blockade, Treg-based therapies, and local biomaterial-enabled immunoprotection.

Looking ahead, the next era of pancreas transplantation research will be defined by the convergence of precision reconditioning and targeted immunomodulation.

**Figure 1:**
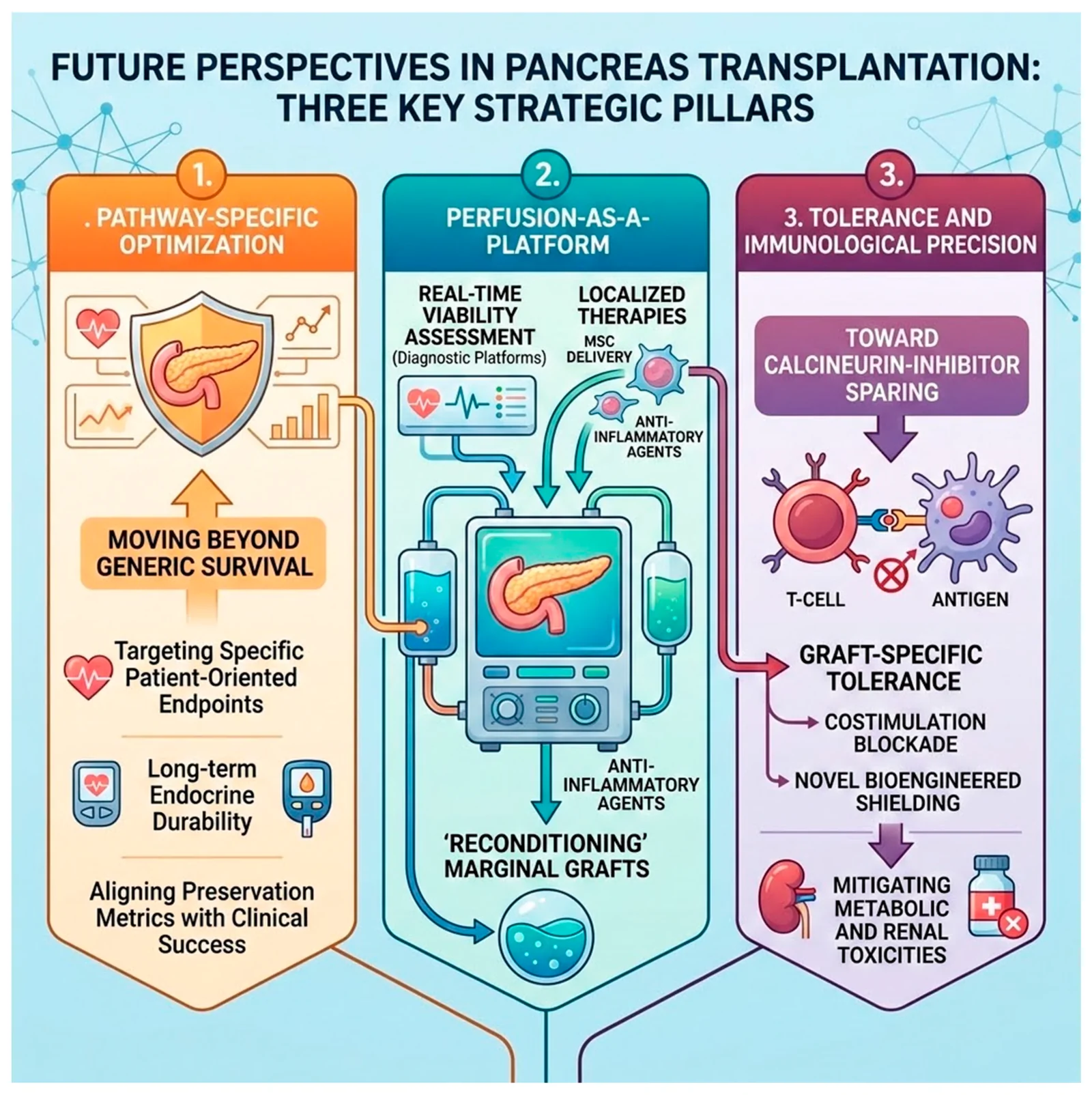
Future perspectives in pancreas transplantation.

In summary, the transition from binary comparisons to a strategic alignment of preservation, application, and immunological precision marks the arrival of a new era in pancreas and islet transplantation. By refining these multi-layered approaches, we move closer to the ultimate goal: stable, long-term glycaemic control that restores patient quality of life while minimizing the burden of chronic systemic therapy (Figure 1).

AI statement: The author(s) declared that generative AI was used in the creation of this manuscript. The authors used Leapspace^®^ to help with the literature search and to structure a first draft of the manuscript. The AI-generated material was rigorously reviewed for accuracy, and the final version was written by the authors. The authors take full responsibility for the content of the publication.

Figure 1 in this article was generated by Gemini^®^, and reasonable efforts have been made to ensure accuracy, including review by the authors wherever possible.

## Figures and Tables

**Table 1:** Main biotech approaches.

Approach	What It Does	Evidence from the Retrieved Set
Costimulation blockade	Targets T-cell activation pathways such as CD28/CD80/CD86 and CD40/CD154 to reduce rejection without conventional CNI exposure [48,49]	CTLA4-Ig/belatacept was engineered to improve potency over earlier CTLA4-Ig constructs, and the literature describes it as a major advance in transplant immunomodulation [48,50,51]. In islet transplantation, CTLA4-Ig-based strategies and related costimulation blockade prolonged graft survival, especially in combination regimens [52,53].
Monoclonal antibodies/induction biologics	Uses depletional or pathway-specific antibodies to reduce early rejection and support insulin independence [54,55]	Daclizumab, anti-CD3, alemtuzumab, anti-CD25, and anti-TNF approaches were used to improve islet outcomes and durability [40,56,57,58,59,60].
Treg therapy and tolerance engineering	Expands or infuses regulatory T cells to shift toward donor-specific tolerance [60,61]	Clinical autologous Treg co-infusion in allogeneic islet transplantation was feasible and safe, and preclinical work showed Tregs can prolong islet graft survival and induce systemic tolerance [42,62,63,64].
Biomaterials and encapsulation	Physically isolates islets while permitting nutrient/oxygen exchange [65,66]	Microencapsulation, macroencapsulation, conformal PEG coating, and hydrogel systems aim to avoid chronic immunosuppression; key limits are hypoxia, fibrosis, and retrieval challenges [67,68,69,70,71].
Local drug delivery/microparticles	Delivers immunosuppressants or cytokines directly at the graft site to reduce systemic toxicity [72]	Local rapamycin release, collagen/drug depots, TGF-β1 scaffolds, VEGF-controlled scaffolds, and NICHE-style localized delivery reduced rejection or improved engraftment while limiting systemic exposure [73,74,75,76,77].
Alternative agents to reduce CNI toxicity	Moves away from tacrolimus-heavy maintenance toward less toxic regimens [78]	The Edmonton shift to steroid-free low-dose tacrolimus/sirolimus improved early outcomes, but later work emphasized CNI-sparing or CNI-free strategies such as belatacept, MMF-based regimens, and antibody induction [26,58,79,80,81,82].

**Table 2:** Timeline of pancreas perfusion developments.

Year	Development	Why It Mattered	Citations
2010	Early hypothermic pulsatile perfusion and 24 h hypothermic machine perfusion (HMP] in porcine pancreas models	Showed ex situ hypothermic perfusion could preserve morphology and support later islet isolation work	[93,94]
2012	Review of persufflation as an organ preservation method	Framed gaseous oxygen perfusion as an alternative oxygenation strategy	[95]
2014	Portal venous oxygen persufflation in a DCD rat pancreas model; ductal perfusion during human islet procurement	Strengthened the case for oxygen delivery and targeted perfusion to improve islet yield and viability	[96,97]
2015	Ex vivo normothermic perfusion used for quality assessment of discarded human pancreases	Marked an early step toward viability testing under near-physiologic conditions	[98]
2018	Translational work moved from porcine to human models, including human donor HMP, human hypothermic pulsatile perfusion, and ex vivo normothermic porcine models	Showed both hypothermic and normothermic platforms were becoming technically feasible in human-relevant systems	[99,100,101,102]
2021	Successful pancreas allotransplantations after HMP in pigs; HOPE feasibility for human islet isolation; NRP outcomes in pancreas transplantation; ex situ normothermic reperfusion for assessment	Major translational phase where HMP, oxygenated perfusion, and NRP moved closer to clinical application	[103,104,105,106]
2022	Clinical comparison of preservation solutions; normothermic ex situ pancreas perfusion for porcine grafts; normothermic allograft preservation before transplant	Expanded both clinical preservation comparisons and experimental normothermic preservation protocols	[107,108,109]
2023	Feasibility study of normothermic ex vivo perfusion in discarded human pancreas allografts; ESOT consensus on pancreas machine perfusion	Signaled increasing clinical interest and standard-setting around machine perfusion	[110,111]
2025–2026	Comparative work on HOPE and continuous vs. end-ischemic oxygenated HMP	Optimization of oxygenated hypothermic protocols	[112,113]

**Table 3:** Key perfusion modalities in pancreas transplantation.

Perfusion Modality	Key Findings	Limitations/Issues	Sources
Static Cold Storage (SCS)	Current clinical standard; UW remains gold standard; acceptable outcomes in DBD donors.	Limited ischemia–reperfusion protection; not sufficient for marginal or DCD grafts.	[107]
Hypothermic Machine Perfusion [HMP)	Feasible in porcine and human grafts; reduces cellular injury; maintains ATP; improves islet yield; preserves histology.	Risk of edema; optimal protocols (pressure/flow, perfusate) not defined [10].	[93]
Oxygenated Hypothermic Perfusion (HOPE)	Improves oxygenation and ATP; reduces succinate accumulation; improves beta-cell function; protective effects enhanced with oxygen carriers (M101).	Clinical studies lacking; protocol variability.	[103,104,113]
Normothermic Machine Perfusion (NMP)	Demonstrated metabolic activity, insulin production, viability, feasibility in human and porcine models.	Significant edema and necrosis in some human studies; unstable protocols.	[108,110]
Normothermic Regional Perfusion (NRP)	Used clinically in DCD donors; may reduce warm ischemia; improves kidney/liver outcomes; feasibility for pancreas under evaluation.	Mixed results for pancreas; pancreas-specific evidence is currently limited to conceptual discussion and requires further study before clinical adoption.	[105,118]
Persufflation (Gaseous O_2_ Perfusion)	Improves islet yield and viability in DCD models; potential alternative oxygenation method.	Historical safety concerns (embolism risk); limited pancreas data.	[95,96]

**Table 4:** Main parameter/biomarker types.

Parameter/Biomarker Class	Clinical Setting	Clinical Meaning
Perfusion imaging	CT blood flow, blood volume, mean transit time, permeability surface area product; CEUS; MRI perfusion; perfusion angiography/ICG	Regional pancreatic perfusion, microcirculatory dysfunction, graft viability, or ischemic injury [119,125,126]
Microcirculation imaging	OPS imaging, laser Doppler flowmetry, laser speckle contrast imaging, handheld/multimodal microcirculation platforms	Capillary perfusion, functional capillary density, perfusion heterogeneity, reperfusion injury [122,125,127,128,129]
Tissue oxygenation	pO2/tissue oxygen tension, NIRS, oxygen saturation, oxygen extraction indicators	Local oxygen delivery/consumption balance and early ischemia [98,120,130,131,132]
Biochemical markers	Lactate, amylase, lipase, LDH, glucose, pyruvate, insulin, C-peptide, CRP, trypsinogen-2, TNF-alpha	Ischemia, anaerobic metabolism, inflammation, exocrine/endocrine graft function, and postoperative complications [120,121,122,124,133]

**Table 5:** Parameters and biomarkers utilized in pancreas machine perfusion and related evidence.

Parameter	What It Predicts	Evidence
Flow/tissue flow rate	Better viability and post-transplant function when higher and stable; low tissue flow rate associated with reperfusion injury [135].	Consistent
Vascular resistance	Lower or falling resistance is associated with better graft quality; stable resistance was used as a viability marker [93,135,137].	Consistent
Perfusion pressure stability	Lower pressures avoided edema; higher pressures cause severe edema in pancreas perfusion models [93,99].	Consistent
Lactate/pH	Maintained pH and falling lactate during normothermic perfusion suggested viability; lactate clearance is a proposed viability marker [98].	Promising, mostly normothermic data
Edema/weight gain	Excess weight gain/edema tracked worse preservation; minimal edema aligned with better graft quality [99,108,110].	Consistent
Amylase/lipase/cell injury markers	Higher amylase signaled injury; amylase, lipase, LDH, and cfDNA were tracked as injury markers, but not all are yet validated predictors [98,113,135].	Useful but not fully standardized
ATP/metabolic markers	ATP is described as a sensitive viability marker and improved with normothermic preservation [110].	Experimental, promising

## Data Availability

The original contributions presented in this study are included in the article. Further inquiries can be directed to the corresponding author.

## Abbreviations

CNI	calcineurin inhibitor
DCD	donation after circulatory death
ECD	extended criteria donor
IRI	ischemia–reperfusion injury
HMP	hypothermic machine perfusion
HOPE	hypothermic oxygenated machine perfusion
NEVP	normothermic ex vivo perfusion
PMP	pancreas machine perfusion
SCS	static cold storage
ROS	reactive oxygen species
NRP	normothermic regional perfusion
